# Supplementary material for: The Alterations of Serum N-glycome in Response to SARS-CoV-2 Vaccination
Source: Int J Mol Sci. 2023 Mar 25;24(7):6203. doi: 10.3390/ijms24076203 (PMC10093923; doi:10.3390/ijms24076203)
Supplement: Supplementary file 1 [file ijms-24-06203-s001.zip › ijms-2296183-supplementary.pdf]

Supplementary

The Alterations of Serum N-glycome in Response to SARS-CoV-2 Vaccination

Dalma Dojcsák[1], Zsófia Kardos[2], Miklós Szabó[2], Csaba Oláh[2], Zsolt Körömi[2], Béla Viskolcz[1] and Csaba Váradi[1*]

[1] Advanced Materials and Intelligent Technologies Higher Education and Industrial Cooperation Centre, University of Miskolc, Hungary 3515;
[2] Borsod Academic County Hospital, Hungary 3526

Supplementary Table S1.

Glycan structures [Theoretical m/z]$^{2+}$		Average area % ± std deviation				Significance level with 95% confidence (* Statistically significant p ≤ 0.05)
		Covid-Vaccine-	Covid-Vaccine+	Covid+Vaccine-	Covid+Vaccine+	
FA2 [841.94] $^{2+}$	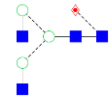	4.28 ± 2.02	3.72 ± 1.10	3.88 ± 1.12	3.63 ± 1.55	0.794
M5 [727.89] $^{2+}$	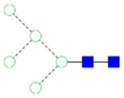	2.55 ± 0.86	2.50 ± 0.41	2.33 ± 0.45	2.20 ± 0.54	0.361
FA2G1 [922.97] $^{2+}$	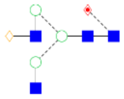	3.67 ± 0.94	3.74 ± 0.88	3.62 ± 0.61	3.37 ± 0.97	0.500
FA2G1 [922.97] $^{2+}$	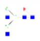	1.74 ± 0.57	1.82 ± 0.48	1.62 ± 0.23	1.62 ± 0.37	0.701
FA2BG1 [1024.51] $^{2+}$	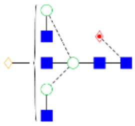	1.33 ± 0.36	1.38 ± 0.27	1.24 ± 0.32	1.20 ± 0.30	0.575
M6 [808.92] $^{2+}$	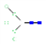	1.32 ± 0.38	1.46 ± 0.28	1.25 ± 0.24	1.25 ± 0.34	0.128
A2G2 [930.97] $^{2+}$	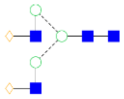	1.15 ± 0.33	1.27 ± 0.21	1.29 ± 0.26	1.20 ± 0.17	0.716
A2BG2 [1032.51] $^{2+}$	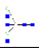	0.51 ± 0.12	0.53 ± 0.09	0.59 ± 0.17	0.50 ± 0.10	0.336

FA2G2 [1004.00] $^{2+}$	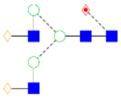	3.04 ± 0.84	3.54 ± 1.07	2.93 ± 0.51	2.93 ± 0.81	0.208
FA2BG2 [1105.54] $^{2+}$	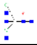	2.33 ± 0.36	2.43 ± 0.27	2.26 ± 0.40	2.18 ± 0.34	0.320
FA2G2S1 [1149.55] $^{2+}$	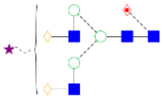	0.40 ± 0.09	0.44 ± 0.07	0.42 ± 0.05	0.39 ± 0.10	0.132
A2G2S1 [1076.52] $^{2+}$	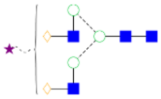	2.94 ± 0.67	3.09 ± 0.52	3.04 ± 0.31	3.03 ± 0.51	0.991
A2G2S1 [1076.52] $^{2+}$	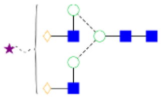	9.43 ± 1.26	9.85 ± 1.10	10.29 ± 1.09	9.64 ± 0.87	0.202
A2FG2S1 [1149.55] $^{2+}$	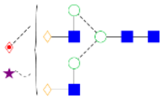	0.69 ± 0.44	0.56 ± 0.24	0.70 ± 0.29	0.56 ± 0.20	0.224
FA2G2S1 [1149.55] $^{2+}$	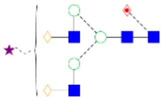	4.36 ± 1.12	4.86 ± 0.74	4.65 ± 0.79	4.61 ± 0.76	0.661
FA2BG2S1 [1251.09] $^{2+}$	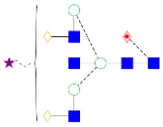	0.69 ± 0.37	0.64 ± 0.13	0.68 ± 0.18	0.63 ± 0.14	0.246
A2G2S2 [1222.06] $^{2+}$	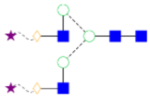	2.73 ± 0.85	3.28 ± 0.70	3.02 ± 1.19	2.90 ± 0.65	0.229
FA2BG2S1 [1251.08] $^{2+}$	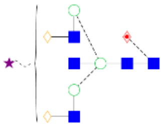	2.16 ± 0.42	2.08 ± 0.73	2.29 ± 0.84	2.06 ± 0.76	0.737
FA2BG2S1 [1251.08] $^{2+}$	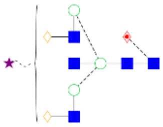	0.72 ± 0.63	0.65 ± 0.08	0.54 ± 0.07	0.53 ± 0.15	*0.013
FA2BG2S1 [1251.08] $^{2+}$	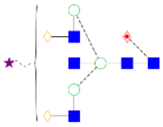	0.46 ± 0.06	0.44 ± 0.06	0.48 ± 2.81	0.47 ± 0.06	0.328
A2G2S2 [1222.06] $^{2+}$	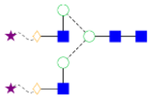	29.87 ± 2.39	29.08 ± 2.14	29.71 ± 0.15	30.02 ± 1.75	0.657

A2G2S2 [1222.06] $^{2+}$	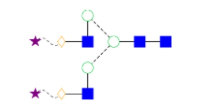	1.07 ± 0.28	1.24 ± 0.17	1.09 ± 0.27	1.22 ± 0.12	*0.027
FA2G2S2 [1295.09] $^{2+}$	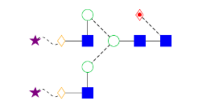	0.60 ± 0.25	0.48 ± 0.13	0.51 ± 1.04	0.37 ± 0.14	*0.001
FA2G2S2 [1295.09] $^{2+}$	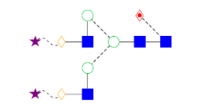	4.11 ± 0.93	3.83 ± 0.94	4.23 ± 0.59	4.28 ± 0.96	0.545
FA2BG2S2 [1396.63] $^{2+}$	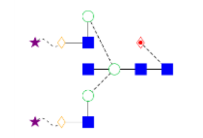	2.38 ± 0.51	2.20 ± 0.55	2.43 ± 0.58	2.33 ± 0.53	0.620
A3G3S2 [1404.63] $^{2+}$	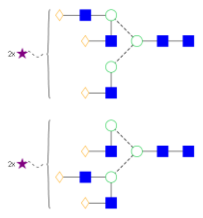	1.58 ± 0.40	1.75 ± 0.41	1.78 ± 0.05	1.86 ± 0.35	0.313
A3G3S2 [1404.63] $^{2+}$	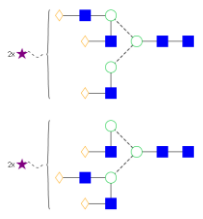	0.20 ± 0.08	0.22 ± 0.06	0.21 ± 0.24	0.28 ± 0.18	0.334
A3G3S2 [1404.63] $^{2+}$	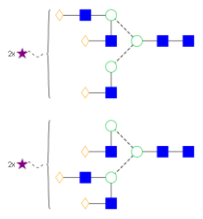	0.92 ± 0.25	1.02 ± 0.28	0.95 ± 0.24	1.10 ± 0.30	0.213
A3G3S2 [1404.63] $^{2+}$	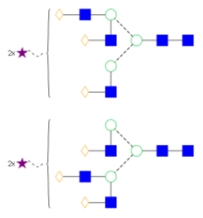	0.53 ± 0.25	0.43 ± 0.09	0.46 ± 0.13	0.57 ± 0.21	0.177
FA3G3S2 [1477.66] $^{2+}$	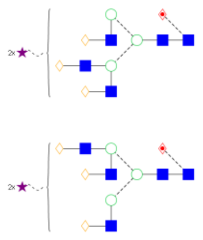	1.14 ± 0.25	1.16 ± 0.22	1.17 ± 0.23	1.10 ± 0.28	0.969

FA3G3S2 [1477.66] 2+	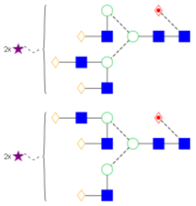	0.38 ± 0.06	0.37 ± 0.08	0.36 ± 0.05	0.42 ± 0.14	0.122
A3G3S3 [1550.18] 2+	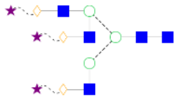	4.32 ± 1.08	4.17 ± 1.22	4.55 ± 1.78	5.01 ± 0.99	0.174
A3G3S3 [1550.18] 2+	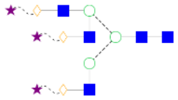	0.32 ± 0.09	0.33 ± 0.08	0.29 ± 0.09	0.35 ± 0.09	0.349
A4G4S2 [1587.20] 2+	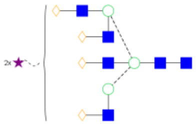	0.33 ± 0.09	0.34 ± 0.10	0.27 ± 0.09	0.39 ± 0.12	*0.027
A4G4S2 [1587.20] 2+	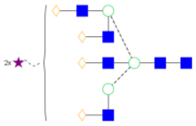	0.44 ± 0.13	0.41 ± 0.14	0.44 ± 0.16	0.53 ± 0.20	0.165
A3G3S3 [1550.18] 2+	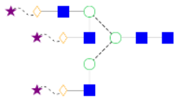	1.53 ± 0.38	1.27 ± 0.31	1.32 ± 0.40	1.69 ± 0.52	*0.045
A3FG3S3 [1623.21] 2+	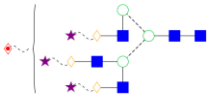	2.23 ± 0.89	2.05 ± 0.69	1.91 ± 0.88	1.97 ± 0.55	0.454
A3G3S3 [1550.18] 2+	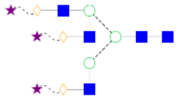	0.76 ± 0.23	0.71 ± 0.22	0.67 ± 0.16	0.96 ± 0.25	*0.009
A4G4S3 [1155.50] 2+	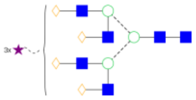	0.24 ± 0.14	0.20 ± 0.09	0.18 ± 0.12	0.17 ± 0.09	0.253
A4G4S3 [1155.50] 2+	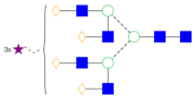	0.29 ± 0.09	0.22 ± 0.07	0.20 ± 0.07	0.30 ± 0.10	*0.001
A4G4S4 [1252.53] 2+	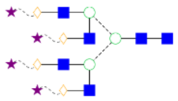	0.27 ± 0.10	0.23 ± 0.16	0.16 ± 0.06	0.20 ± 0.08	*0.049

Supplementary Figure S1. Fragmentation of parent ion: A2G2S2 [2443.2]+

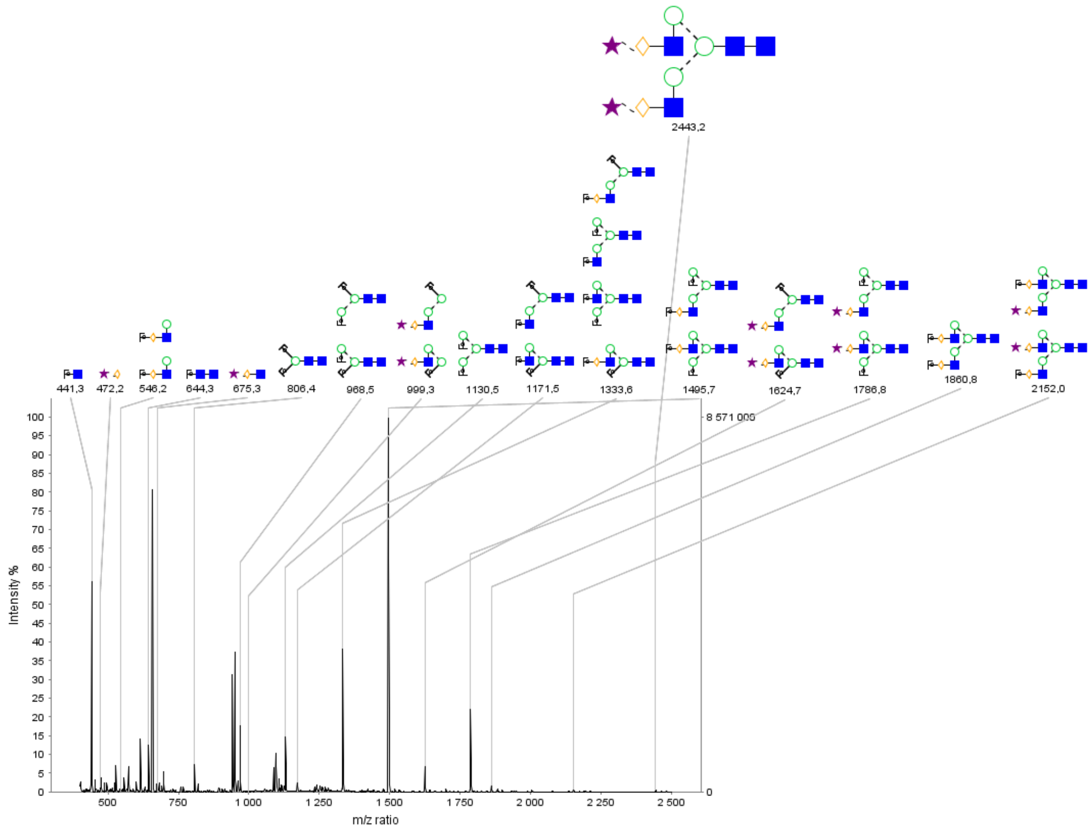

Supplementary Figure S2. Fragmentation of parent ion: FA2G2S2 [2589.2]+

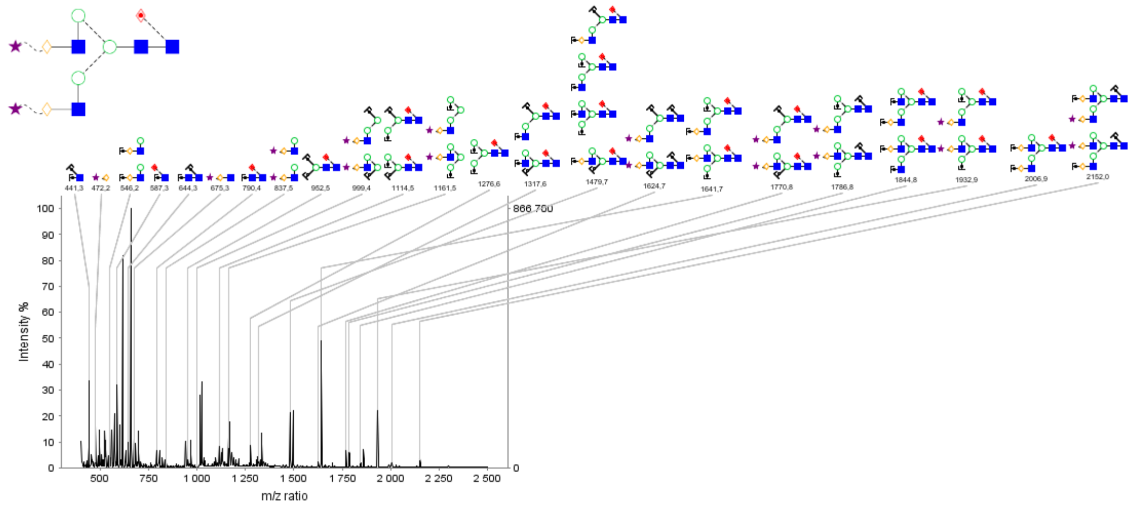

Supplementary Figure S3. Fragmentation of parent ion: A2G2S1 [2152.0]+

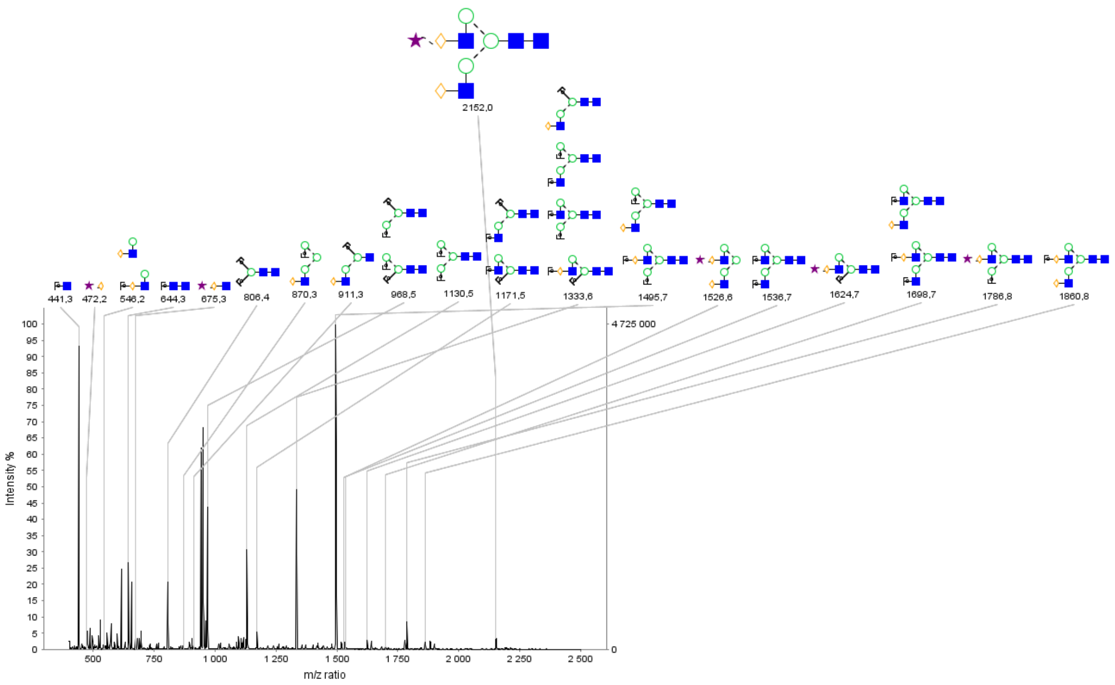

Supplementary Figure S4. Fragmentation of parent ion: FA2 [1682.7]+

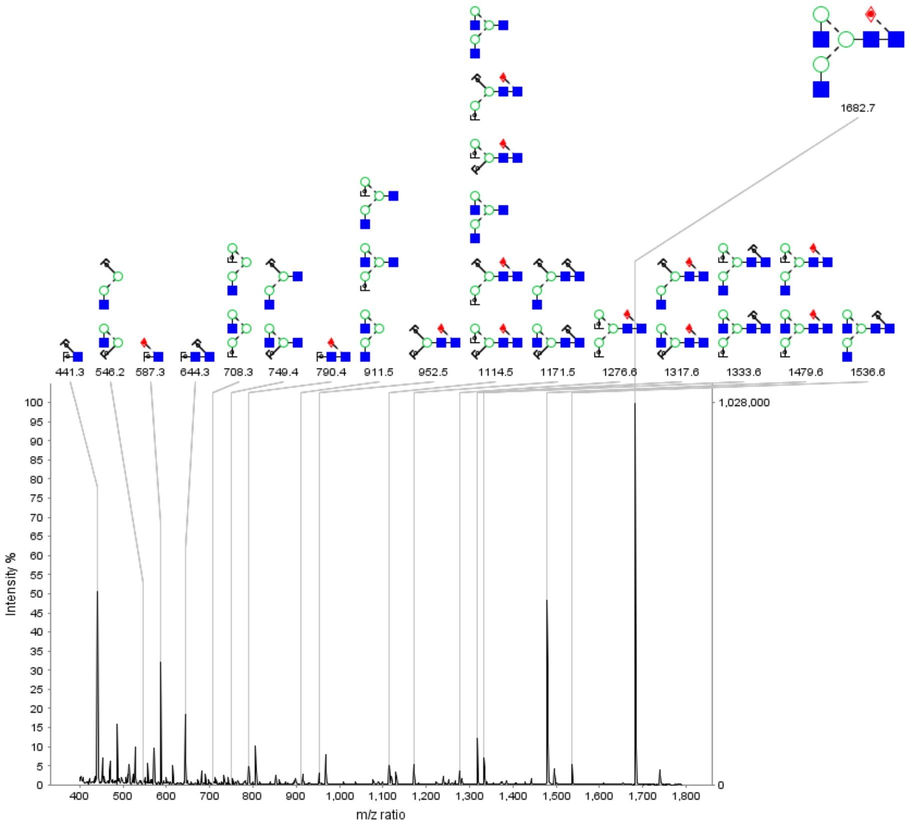

Supplementary Figure S5. Fragmentation of parent ion: A3G3S3 [3099.3]⁺

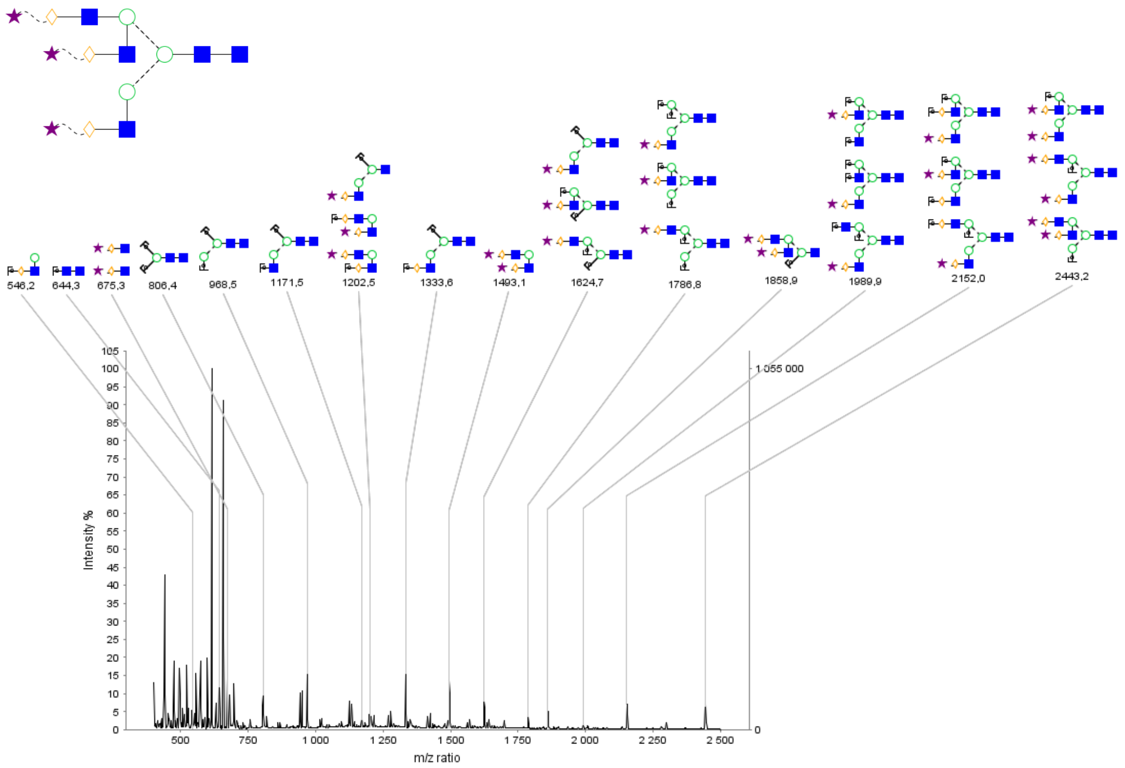

Supplementary Figure S6. Fragmentation of parent ion: M5 [1454.6]⁺

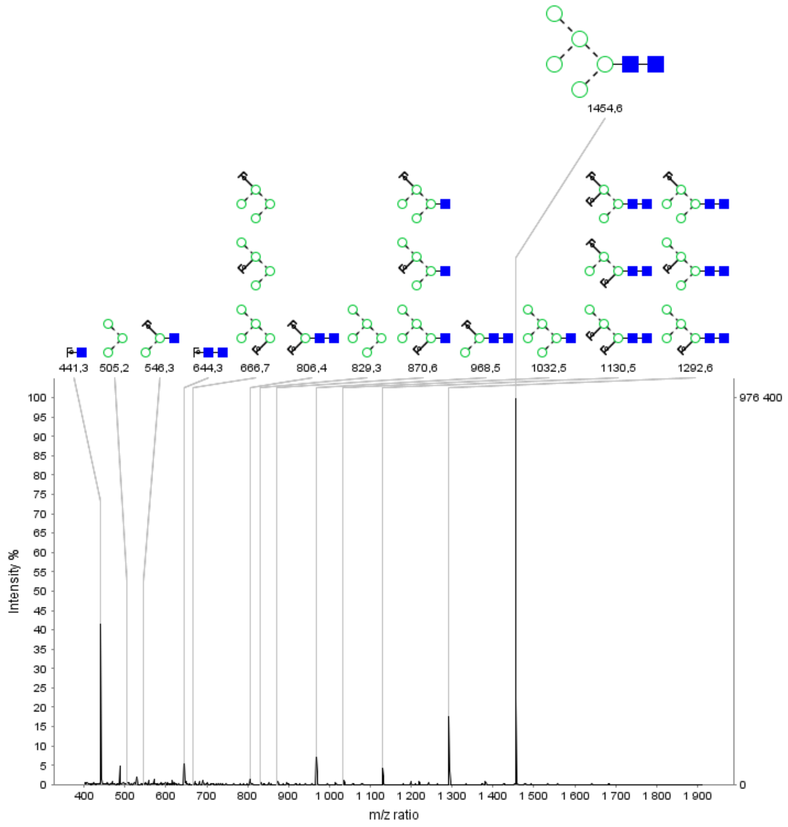

Supplementary Figure S7. Roc curve analysis of A2G2S2 (A), FA2G2S2 (B) and their significance levels (C)

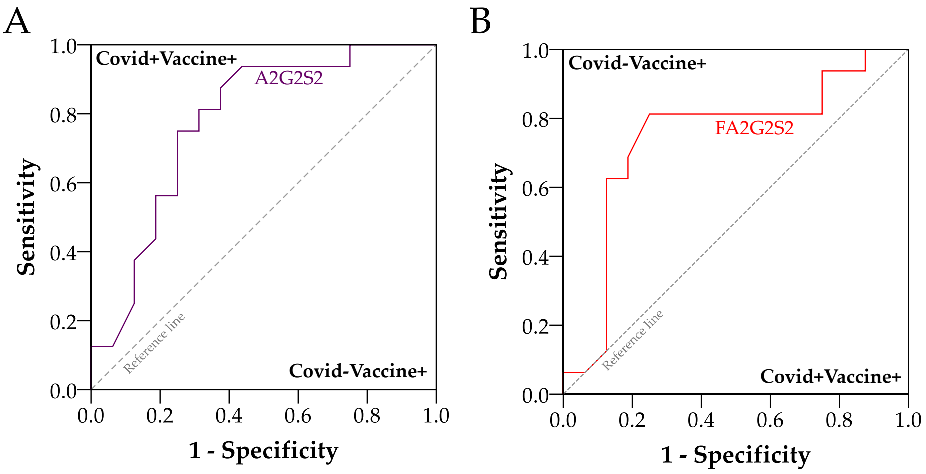

C

Area Under the Curve

Test variable	Area	Std. Error[a]	Asymptotic Sig.[b]	Asymptotic 95% Confidence Interval	
				Lower Bound	Upper Bound
A2G2S2	0.78	0.09	0.01	0.61	0.94
FA2G2S2	0.74	0.09	0.02	0.56	0.93

a. Under the nonparametric assumption

b. Null hypothesis: true area = 0.5
